# Platelet-to-lymphocyte and Neutrophil-to-lymphocyte Ratios Predict Target Vessel Restenosis after Infrainguinal Angioplasty with Stent Implantation

**DOI:** 10.3390/jcm9061729

**Published:** 2020-06-03

**Authors:** Silvia Lee, Timothy Hoberstorfer, Patricia P. Wadowski, Christoph W. Kopp, Simon Panzer, Thomas Gremmel

**Affiliations:** 1 Department of Internal Medicine II, Medical University of Vienna, 1090 Vienna, Austria; silvia.lee@meduniwien.ac.at (S.L.); n11700883@students.meduniwien.ac.at (T.H.); patricia.wadowski@meduniwien.ac.at (P.P.W.); christoph.kopp@meduniwien.ac.at (C.W.K.); 2 Department of Blood Group Serology and Transfusion Medicine, Medical University of Vienna, 1090 Vienna, Austria; simon@wsn.at; 3 Department of Internal Medicine, Cardiology and Nephrology, Landesklinikum Wiener Neustadt, 2700 Wiener Neustadt, Austria; 4 Institute of Vascular Medicine and Cardiac Electrophysiology, Karl Landsteiner Society, 3100 St. Poelten, Austria

**Keywords:** peripheral artery disease, ratios, target vessel restenosis, target vessel reocclusion, platelet reactivity, neutrophil-platelet aggregates

## Abstract

Platelet-to-lymphocyte (PLR), neutrophil-to-lymphocyte (NLR) and lymphocyte-to-monocyte (LMR) ratios are associated with the occurrence of critical limb ischemia in peripheral artery disease (PAD). We therefore investigated whether PLR, NLR or LMR are linked to target vessel restenosis (TVR) following infrainguinal angioplasty and stenting. Moreover, we studied on-treatment platelet reactivity and neutrophil-platelet aggregate (NPA) formation as potential underlying mechanisms. Platelet, neutrophil, lymphocyte and monocyte counts were determined one day after angioplasty and stenting in 95 stable PAD patients. Platelet reactivity and NPA formation in response to protease-activated receptor−1 stimulation were measured by light transmission aggregometry (LTA) and flow cytometry, respectively. PLR and NLR were significantly higher in patients who subsequently developed TVR (both *p* < 0.05). In contrast, LMR did not differ significantly between patients without and with TVR (*p* = 0.28). A PLR ≥ 91 and NLR ≥2.75 were identified as the best thresholds to predict TVR, providing sensitivities of 87.5% and 81.3%, and specificities of 34.9% and 50.8%, respectively, and were therefore defined as high PLR and high NLR. TVR occurred significantly more often in patients with high PLR and high NLR than in those with lower ratios (both *p* < 0.05). Patients with high PLR and high NLR exhibited significantly increased on-treatment platelet aggregation compared to those with lower ratios, and patients with high PLR had higher levels of NPA formation (all *p* < 0.01). In conclusion, PLR and NLR predict TVR after infrainguinal angioplasty with stent implantation. Platelet activation and neutrophil-platelet interaction may be involved in the underlying pathomechanisms

## 1. Introduction

Peripheral artery disease (PAD) is a frequent manifestation of atherosclerosis, which is accompanied by a poor prognosis, in particular in more advanced stages of the disease [1]. Treatment options range from controlling cardiovascular risk factors and antithrombotic therapy in asymptomatic patients to endovascular or surgical revascularization in symptomatic PAD [2]. Patients undergoing infrainguinal angioplasty and stenting frequently suffer target vessel restenosis (TVR) [3]. Besides established cardiovascular risk factors, easy-accessible laboratory parameters may be of use to optimize risk stratification in PAD. Indeed, several markers have been associated with the occurrence of ischemic outcomes after endovascular interventions for PAD [4,5,6]. For instance, low levels of serum cholinesterase and high levels of α-hydroxybutyrate dehydrogenase were linked to an increased risk of TVR and atherothrombotic outcomes, respectively [5,6]. Moreover, recent studies reported that platelet-to-lymphocyte (PLR), neutrophil-to-lymphocyte (NLR) and lymphocyte-to-monocyte (LMR) ratios were able to predict the occurrence of critical limb ischemia in PAD [7,8,9]. In detail, increased PLR and NLR as well as decreased LMR were associated with critical limb ischemia. In this regard, platelet and leukocyte activation and in particular their interaction with one another might foster the development and progression of atherosclerosis and thrombosis [10]. As previously shown, activated neutrophils can induce platelet activation [11,12]. Furthermore, neutrophil and platelet activation as well as neutrophil-platelet aggregate (NPA) formation were linked to ischemic outcomes in cardiovascular disease [3,13,14,15]. Since critical limb ischemia is a frequent consequence of TVR, we sought to investigate whether PLR, NLR or LMR are linked to TVR following elective infrainguinal angioplasty and stenting. In addition, we studied on-treatment platelet reactivity and neutrophil-platelet aggregate (NPA) formation to reveal potential underlying mechanisms.

## 2. Patients and Methods

### 2.1. Patients

We included 95 patients who underwent successful infrainguinal angioplasty with stent implantation at the Division of Vascular Medicine of the Medical University of Vienna. All patients had PAD with Rutherford stages of 2–3, i.e., intermittent claudication, due to infrainguinal artery stenosis or occlusion as assessed by color-coded sonography. All patients had been on low-dose aspirin (100 mg/day) for at least two weeks prior to angioplasty, and received 75 mg of clopidogrel per day for three months following the intervention.

Exclusion criteria were a therapy with vitamin K antagonists (VKA) or non-VKA oral anticoagulants, a known intolerance to aspirin or clopidogrel, the prescription of dipyridamole, nonsteroidal anti-inflammatory drugs or ticlopidine, known bleeding disorders, hematologic malignancies, heparin-induced thrombocytopenia, severe hepatopathy, known qualitative platelet defects, a haematocrit < 30%, a thrombocyte count < 100.000 or > 450.000/µL and major surgery within one week prior to study inclusion. 

The Ethics Committee of the Medical University of Vienna (Project identification code: EK 126/2007; date of approval: 20 November 2007) approved the study protocol in accordance with the Declaration of Helsinki. We obtained written informed consent from all study participants.

### 2.2. Blood Sampling

As previously described, all blood samples were taken by the same person by aseptic venipuncture one day after the angioplasty procedure [16]. The respective investigator applied only a light tourniquet, which was released immediately in order to avoid procedural deviations of the assessed variables.

### 2.3. Measurement of Platelet, Neutrophil, Lymphocyte and Monocyte Count 

Platelet, neutrophil, lymphocyte and monocyte counts were measured in the central laboratory of the Medical University of Vienna according to standardized protocols.

### 2.4. Light transmission Aggregometry

The APACT 4S Plus aggregometer (LABiTec, Ahrensburg, Germany) was used for LTA as previously published [17]. Platelet rich plasma (PRP) was obtained by centrifugation of citrate-anticoagulated whole blood at 150× *g* for 10 min at room temperature. Subsequently, platelet poor plasma (PPP) was generated by re-centrifugation of the remaining specimen at 2000× *g* for 10 min. The light transmittance of PPP was considered as 100% aggregation. Thrombin receptor-activating peptide (TRAP; 25 µM; Rolf Greiner BioChemica, Flacht, Germany) was used as agonist to initiate platelet aggregation. Maximal aggregation % was calculated by comparing the increase in light transmittance through PRP after addition of TRAP to the optical density of PPP.

### 2.5. Neutrophil-platelet Aggregate (NPA) Formation

NPA were measured as previously published with little modification [18]. In brief, TRAP (7.1 μM) was added as platelet agonist to 5 µL whole blood mixed with 55 µL HEPES-buffered saline. After an incubation of 10 min, the monoclonal antibodies anti-CD45-peridinin chlorphyll protein (clone 2D1, BD) and anti-CD41-phycoerythrin (clone P2, Immunotech) or istoype-matched controls were added. After 15 min, the samples were diluted with FACSlysing solution and at least 10,000 CD45+ events were acquired immediately. Neutrophils were identified within these events based on their side scatter versus CD45 characteristics. Subsequently, the neutrophil population was analyzed for CD45 + CD41+ and CD45 + CD41-events. The percentage of CD45 + CD41+ events was recorded as NPA.

### 2.6. Clinical Endpoints

The occurrence of study endpoints was assessed via telephone calls and at follow-up visits of the study participants to our outpatient department. TVR > 80% as measured by colour-coded duplex sonography within 2 years after the percutaneous intervention was defined as the primary endpoint. The composite of the first occurrence of transient ischemic attack (TIA) or nonfatal stroke, nonfatal myocardial infarction, and cardiovascular death within two years was defined as the secondary endpoint.

### 2.7. Sample Size Calculation and Statistical Analysis

We based our sample size calculation on the mean ± SD (3.39 ± 1.12) of NLR in 30 stable patients with PAD (16 female, 14 male; age 66 years (61–73 years)) who had undergone peripheral angioplasty and stenting 24 h before [19]. Thereby, with a two-year adverse event rate of 30% we had to enrol 90 patients to be able to detect a 25% relative difference of NLR between patients without and with the primary endpoint with a power of 92% (using a two-sided alpha level of 0.05). We included 5 additional patients to compensate for potential loss to follow-up.

The Statistical Package for Social Sciences (IBM SPSS version 26, Armonk, New York, USA) was used for all analyses. Continuous variables were given as the median (interquartile range), categorical variables were provided as a number (%). Mann Whitney U tests were applied to detect differences in continuous variables. Differences in categorical variables were detected with the chi-square test and the Fisher’s exact test, as appropriate. Spearman rank correlation was performed to assess correlations of PLR, NLR and LMR with high-sensitivity C-reactive protein (hsCRP). The ability of PLR and NLR to distinguish between patients without and with TVR was determined by receiver operating characteristic (ROC) curve analyses. The respective *p*-values were calculated with the DeLong test. PLR and NLR values that provided the greatest sum of sensitivity and specificity were considered as optimal cut-off values. The Kaplan-Meier method was used to generate survival curves, and the difference between the groups was assessed with the log-rank test. Cox regression analysis was used to adjust for diabetes, hypertension, hyperlipidemia and smoking. Two-sided *p*-values < 0.05 were considered statistically significant. 

## 3. Results

Characteristics of the overall study population and of patients without and with the primary endpoint are given in Table 1. 

Patient characteristics did not differ significantly between patients without and with TVR within two-year follow-up (Table 1; all *p* > 0.1). Lesions were located in the proximal, proximal and mid, mid, mid and distal, and distal superficial femoral artery in 10, 18, 41, 17, and 9 patients, respectively. TASC A, B and C lesions were seen in 15, 56 and 24 patients, respectively. Median lesion length was 100 mm (50–180 mm) and 2 stents (1–2 stents) were implanted per lesion. All patients received self-expanding bare metal stents. In detail, Protege Everflex (Covidien, Dublin, Ireland), Absolute (Abbott Vascular, Illinois, USA), Astron Pulsar (Biotronik, Berlin, Germany), Xpert (Abbott Vascular, Illinois, USA), Smart (Cordis, Fremont, California, USA) and Epic (Boston Scientific, Marlborough, Massachusetts, USA) stents were implanted in 23, 21, 19, 19, 9 and 4 patients, respectively. No or only mild lesion calcification was seen in all patients, and all patients had at least one patent artery without significant stenosis to the ankle. PLR, NLR and LMR in the overall study population were 108.8 (88.2–159.5), 3.1 (2.4–4.1), and 2.5 (1.9–3.2) respectively. 

**Figure 1:**
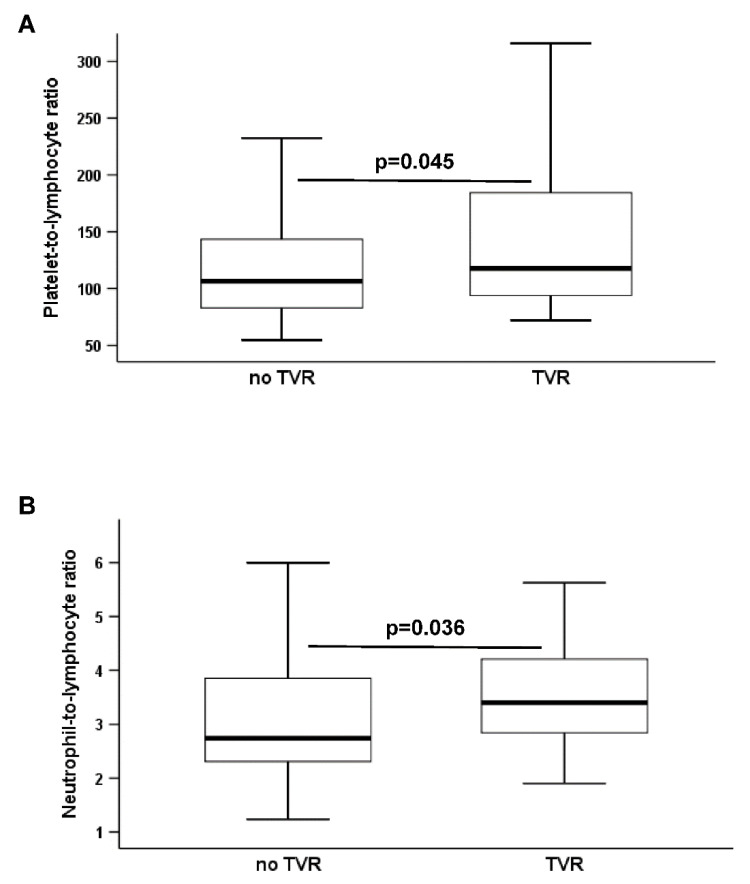
(**A**) Platelet-to-lymphocyte ratio and (**B**) neutrophil-to-lymphocyte ratio in patients without and with the primary endpoint. The boundaries of the box show the lower and upper quartile of data, and the line inside the box represents the median. Whiskers are drawn from the edge of the box to the highest and lowest values that are outside the box but within 1.5 times the box length.

The primary endpoint occurred in 32 patients (33.7%). Of note, no stent fractures and stent thromboses were observed during the two-year follow-up. PLR and NLR at study inclusion were significantly higher in patients who subsequently developed TVR (PLR: 117.7 (94–184.3) vs. 106.5 (82.7–146.8); NLR: 3.4 (2.8–4.2) vs. 2.7 (2.3–4); both *p* < 0.05; Table 2; Figure 1). In contrast, LMR did not differ significantly between patients without and with the primary endpoint (LMR: 2.5 (1.8–3) vs. 2.8 (2–3.3), *p* = 0.28; Table 2).

**Figure 2:**
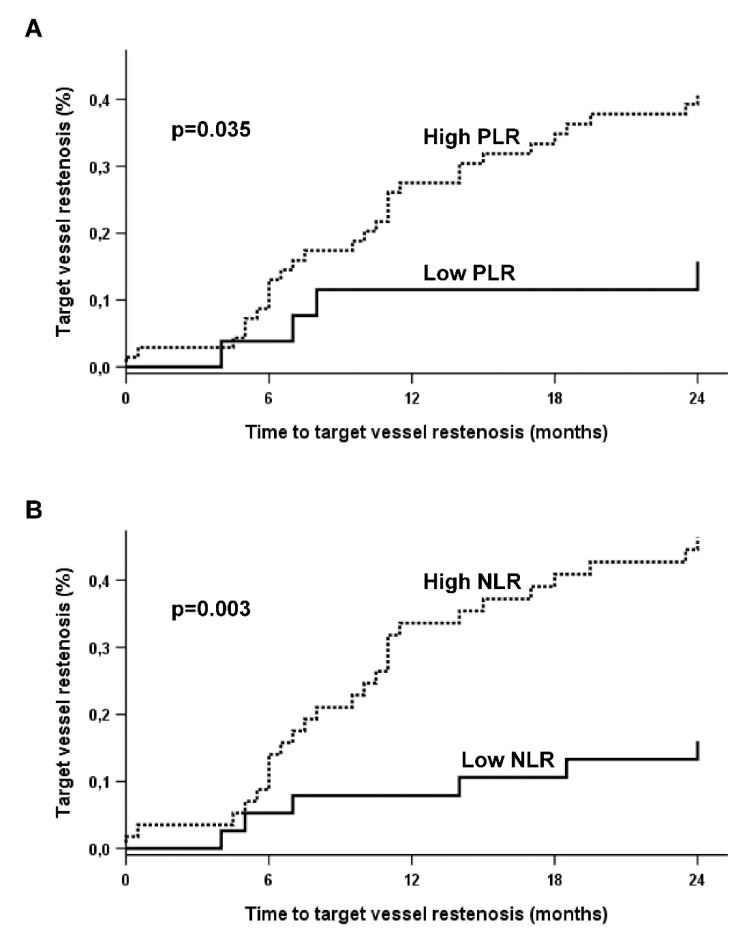
Kaplan-Meier analyses for the cumulative incidence of the primary endpoint in patients with (**A**) high vs. low platelet-to-lymphocyte ratio (PLR), and (**B**) high vs. low neutrophil-to-lymphocyte ratio (NLR). High PLR and high NLR are indicated by the dotted lines, low PLR and low NLR are indicated by the solid lines.

ROC curve analyses revealed that PLR and NLR could distinguish between patients without and with future TVR, with areas under the ROC curves of 0.626 and 0.632 for PLR and NLR, respectively (both *p* < 0.05). A PLR ≥ 91 and NLR ≥ 2.75 were identified as the best thresholds to predict TVR, providing sensitivities of 87.5% and 81.3%, and specificities of 34.9% and 50.8%, respectively, and were therefore defined as high PLR and high NLR. High PLR and high NLR were seen in 69 (72.6%) and 57 (60%) patients, respectively. TVR occurred significantly more often in patients with high PLR and high NLR than in patients with lower ratios (both *p* < 0.05; Figure 2). 

High PLR and high NLR remained significantly associated with TVR after adjustment for diabetes, hypertension, hyperlipidemia and smoking by multivariable Cox regression analysis (Table 3). In detail, high PLR and high NLR were associated with a 3-fold (95% CI 1.1–8.5, *p* = 0.04) and 3.1-fold (95% CI 1.3–7.7, *p* = 0.01) increased risk of TVR, respectively.

The secondary endpoint occurred in 7 patients (7.4% of the study population) within 2 years after the endovascular procedure and comprised 3 nonfatal myocardial infarctions, 2 nonfatal strokes, 1 TIA and 1 cardiovascular death. PLR, NLR and LMR did not differ significantly between patients without and with atherothrombotic events (Table 2; all *p* > 0.5). 

NLR showed a significant positive correlation with hsCRP (r = 0.3, *p* = 0.003), whereas PLR and LMR did not correlate with hsCRP (PLR: r = 0.13, *p* = 0.2; LMR: r = −0.11, *p* = 0.27). Patients with high PLR and high NLR exhibited significantly increased on-treatment platelet reactivity by LTA compared to those with lower ratios (PLR: 70.1% (60.5–75.7%) vs. 53.1% (47–62.6%); *p* = 0.001; NLR: 70.2% (60.2–77.3%) vs. 59.8% (49.6–71.2%); *p* = 0.006), and patients with high PLR had higher levels of NPA (17.7% (10.7–28.9%) vs. 10.4% (7.9–17.5%); *p* = 0.009). Of note, platelet reactivity and NPA formation in response to TRAP did not differ significantly between patients without and with TVR (both *p* > 0.05).

## 4. Discussion

The present study is the first to investigate the associations of PLR, NLR and LMR with TVR following elective infrainguinal angioplasty with stent implantation for symptomatic PAD. High PLR and NLR were linked to an increased risk of TVR over two years. In contrast, LMR did not differ significantly between patients without and with the primary endpoint. None of the investigated ratios was able to predict the occurrence of atherothrombotic events. On-treatment platelet reactivity was significantly increased in patients with high PLR and high NLR compared to patients with lower ratios. Moreover, patients with high PLR exhibited increased NPA formation compared to patients with lower PLR.

In line with our findings, Turak et al. reported an association of NLR with in-stent restenosis following percutaneous coronary intervention (PCI) with bare metal stent implantation [20]. In their study, an NLR >2.73 predicted TVR with a sensitivity of 80% and a specificity of 75%. Thereby, their cut-off was almost identical to the threshold for high NLR obtained in our study. Since all patients in both studies received bare metal stents, it remains to be established if NLR is able to predict TVR after drug eluting stent implantation. In the Ludwigshafen Risk and Cardiovascular Health (LURIC) study, NLR was independently associated with cardiovascular mortality [21]. Moreover, others found NLR to predict mortality in patients with an acute coronary syndrome [22]. Both, high PLR and high NLR were associated with a higher risk of critical limb ischemia in PAD [8,9], and with a higher risk of limb amputation [23,24]. In addition, increased PLR and NLR were linked to a poor prognosis in patients with gastrointestinal cancer [25,26]. Together with our observations, these previous reports underline the biomarker potential of PLR and NLR in various diseases. 

The lack of a significant association of PLR and NLR with atherothrombotic events in our study may be explained by the underlying pathomechanisms. While myocardial infarction, ischemic stroke and TIA frequently result from intravascular platelet activation or thromboembolism with subsequent vessel occlusion, TVR occurs mainly due to intimal hyperplasia on the basis of chronic inflammation. The latter may be reflected by PLR and NLR in this stable cohort despite otherwise normal inflammatory markers. Of note, NLR correlated significantly with hsCRP, whereas no correlations of PLR and LMR with hsCRP were observed. One may therefore speculate that in particular NLR mirrors subclinical inflammation. Indeed, neutrophils play a central role as mediators and amplifiers of inflammation [27]. Furthermore, they release neutrophil-extracellular traps (NETs), which are able to fight bacteria, but may also exert prothrombotic and proinflammatory effects [11,28,29]. In line with these findings, we and others have previously shown associations between markers of NET formation and adverse ischemic events in cardiovascular disease [13,14]. Finally, neutrophils directly interact with platelets via the P-selectin–P-selectin glycoprotein ligand (PSGL)−1 axis as well as via the adhesion molecules glycoprotein (GP) Ib and macrophage−1 antigen [30]. These physical interactions between neutrophils and platelets are promoted and stabilized by several other receptor-ligand interactions such as GPVI and extracellular matrix metalloproteinase inducer (EMMPRIN) or intercellular adhesion molecule 2 (ICAM−2) and lymphocyte function-associated antigen (LFA)−1 [30], leading to the formation of stable NPA [31]. In addition, neutrophils can release antimicrobial peptides from their azurophilic granules which act as platelet agonists [18]. In turn, activated platelets enhance NET formation, and promote neutrophil rolling in the sub-endothelial space via GPIb and GPIIb/IIIa [32,33,34]. Neutrophil activation and transmigration are mediated through the interaction of platelet P-selectin with neutrophil PSGL−1 and the other aforementioned mechanisms [30,31,35]. In summary, neutrophils, platelets and especially their interaction with one another may foster the development and progression of atherosclerosis [27,36]. In this regard, the procoagulant state of platelets might also contribute to TVR [37]. This hypothesis was supported by our findings of increased platelet reactivity in patients with high PLR and high NLR, and increased levels of NPA in patients with high PLR. On the other hand, a high lymphocyte count has previously been associated with limb salvage in critical limb ischemia [38], a finding that may be explained by the mediation of collateral vessel growth by lymphocytes via the secretion of interleukin−16 [39]. Accordingly, high PLR and high NLR may mirror a predominance of proinflammatory and prothrombotic factors over vasoprotective influences, which may account for the higher risk of TVR in the respective patients. 

Besides the above-discussed hypothesis, the low rate of atherothrombotic outcomes in the investigated study population made it difficult to reveal significant differences of PLR, NLR and LMR between patients without and with these events. Since all patients had two follow-up visits at the outpatient department per year and were prescribed state-of-the-art lipid-lowering, antihypertensive and antiplatelet therapy, stringent risk factor management following the angioplasty procedures may have prevented further thrombotic events despite manifest atherosclerosis.

LTA is the historical gold standard of platelet function testing and its results have repeatedly been associated with ischemic outcomes post PCI [40]. NPA are a sensitive marker of platelet activation and reflect the extent of neutrophil-platelet interaction [18,41]. TRAP is a strong protease activated receptor−1 agonist leading to pronounced platelet activation despite dual antiplatelet therapy [42,43]. Moreover, we have previously shown that TRAP inducible platelet surface expression of P-selectin and activated glycoprotein IIb/IIIa are associated with ischemic events after peripheral angioplasty and stenting [3]. In the current study, however, platelet aggregation and NPA formation in response to TRAP did not differ between patients without and with TVR. This may be due to differences in the endpoint definition, the smaller sample size and the use of other parameters of platelet function compared to the previous study. 

A limitation of our study was its rather small sample size, and the areas under the ROC curves of 0.626 and 0.632 for PLR and NLR, respectively, cannot be considered as strong classifiers. We included only PAD patients in a stable clinical condition who underwent scheduled angioplasty with stent implantation due to intermittent claudication. Accordingly, our results cannot be extrapolated to patients with critical limb ischemia. Differences in PLR and NLR may be attributable to individual reactions to the peripheral interventions, and both ratios may have been increased in some patients before the endovascular procedures. Moreover, pre-activation of circulating immune cells and platelets might play a role in the observed findings. Since we only assessed cell counts as well as NPA formation and platelet aggregation 24 h after the intervention, we cannot provide preprocedural values or information on the variability of these parameters over time. The measurement time point was chosen because (A) 1 day after angioplasty, all patients were still at the inpatient ward, and (B) we sought to determine if a single measurement of the ratios may be used for risk stratification. As a next step, it would be important to study platelets and neutrophils isolated from peripheral blood and to provide additional flow cytometry analyses. Furthermore, it would be interesting to investigate if differences in the bioreactivity and endothelialization of different stent types influence platelet and leukocyte activation [44]. Finally, in vitro stenosis models could be of use to address the role of PLR and NLR in the development of TVR.

In conclusion, PLR and NLR predict TVR after infrainguinal angioplasty with stent implantation. Platelet activation and neutrophil-platelet interaction may be involved in the underlying pathomechanisms.

## Figures and Tables

**Table 1:** Clinical, laboratory and procedural characteristics of the overall study population, and of patients. without and with target vessel restenosis (TVR).

Characteristics	Overall (*n* = 95)	noTVR (*n* = 63)	TVR (*n* = 32)	*p*
Demographics				
Age, years	65 (58–74)	64 (58–72)	67 (60–79)	0.14
Male sex, *n* (%)	57 (60)	41 (65.1)	16 (50)	0.16
Body mass index, kg/m^2^	26.5 (24.5–29.1)	27.4 (24.8–29.4)	25.5 (23.9–28.3)	0.14
Medical history				
Hypertension	88 (92.6)	58 (92.1)	30 (93.8)	1
Hyperlipidemia	89 (93.7)	61 (96.8)	28 (87.5)	0.18
Diabetes mellitus	34 (35.8)	19 (30.2)	15 (46.9)	0.11
Active smoking	42 (44.2)	31 (49.2)	11 (34.4)	0.17
Previous myocardial infarction	18 (18.9)	14 (22.2)	4 (12.5)	0.25
Coronary artery disease	31 (32.6)	24 (38.1)	7 (21.9)	0.11
Cerebrovascular disease	22 (23.2)	16 (25.4)	6 (18.8)	0.47
Laboratory data				
Haemoglobin, g/dL	13.7 (12.6–14.7)	13.8 (12.6–14.9)	13.5 (11.9–14.2)	0.34
Haematocrit, %	40.5 (37.1–43.1)	41.2 (37.5–43.8)	39.3 (36.8–41.3)	0.19
White blood cell count, G/L	8.9 (7–10.3)	9.2 (6.8–10.3)	8.7 (7.2–10.1)	0.73
Platelet count, G/L	212 (183–250)	210 (168–253)	221 (203–249)	0.14
Serum creatinine, mg/dL	1 (0.9–1.2)	1 (0.9–1.1)	1.1 (0.9–1.2)	0.55
High-sensitivity CRP, mg/dL	1 (0.3–1.8)	1.1 (0.4–1.8)	0.8 (0.3–1.6)	0.55
Procedure				
Stent implantation	95 (100)	63 (100)	32 (100)	1
Number of stents/patient	2 (1–2)	2 (1–2)	2 (1–2)	0.73
Medication pre-intervention				
Clopidogrel	95 (100)	63 (100)	32 (100)	1
Aspirin	95 (100)	63 (100)	32 (100)	1
Statins	86 (90.5)	59 (93.7)	27 (84.4)	0.16
ACE inhibitors/ARB	81 (85.3)	52 (82.5)	29 (90.6)	0.37
Beta blockers	58 (61.1)	39 (61.9)	19 (59.4)	0.81

Continuous data are shown as median (interquartile range). Dichotomous data are shown as *n* (%). ACE, angiotensin converting enzyme; ARB, angiotensin receptor blockers; CRP, C-reactive protein.

**Table 2:** Platelet-to-lymphocyte (PLR), neutrophil-to-lymphocyte (NLR) and lymphocyte-to-monocyte ratios. (LMR) in patients without and with target vessel restenosis (TVR), and in patients without and with an atherothrombotic event (AE) within 2-year follow-up.

**Ratio**	**No TVR (*n* = 63)**	**TVR (*n* = 32)**	***p***
PLR	106.5 (82.7–146.8)	117.7 (94–184.3)	0.045
NLR	2.7 (2.3–4)	3.4 (2.8–4.2)	0.036
LMR	2.5 (1.8–3)	2.8 (2–3.3)	0.28
**Ratio**	**No AE (*n* = 88)**	**AE (*n* = 7)**	***p***
PLR	108.4 (89.1–155)	150.6 (76.5–172.5)	0.79
NLR	3.1 (2.4–4.2)	2.8 (2.3–4)	0.62
LMR	2.5 (1.8–3.1)	3 (2–3.6)	0.52

**Table 3:** Adjusted hazard ratios, 95% confidence intervals, and *p*-values of multivariable Cox regression analysis.

**(A)**			
**Variable**	**Adjusted Hazard Ratio**	**95% Confidence Interval**	***p*-Value**
High PLR	3	1.1–8.5	0.04
Diabetes	1.7	0.8–3.5	0.16
Hypertension	0.6	0.1–2.7	0.53
Hyperlipidemia	0.4	0.1–2.3	0.12
Smoking	0.6	0.3–1.3	0.23
**(B)**			
**Variable**	**Adjusted Hazard Ratio**	**95% Confidence Interval**	***p*-Value**
High NLR	3.1	1.3–7.7	0.01
Diabetes	1.5	0.7–3.1	0.27
Hypertension	0.7	0.2–3	0.62
Hyperlipidemia	0.5	0.2–1.5	0.23
Smoking	0.6	0.3–1.4	0.24

(**A**) Adjusted hazard ratios, 95% confidence intervals, and *p*-values of multivariable Cox regression analysis of high platelet-to-lymphocyte ratio (PLR), diabetes, hypertension, hyperlipidemia and smoking for target vessel restenosis. (**B**) Adjusted hazard ratios, 95% confidence intervals, and *p*-values of multivariable Cox regression analysis of high neutrophil-to-lymphocyte ratio (NLR), diabetes, hypertension, hyperlipidemia and smoking for target vessel restenosis.
